# Discovering the sluggishness of triathlon running - using the attractor method to quantify the impact of the bike-run transition

**DOI:** 10.3389/fspor.2022.1065741

**Published:** 2022-12-16

**Authors:** Christian Weich, Valentin Barth, Nikolai Killer, Veronica Vleck, Julian Erich, Tobias Treiber

**Affiliations:** ^1^ Sports Science Department, University of Konstanz, Konstanz, Germany; ^2^ Physics Department, University of Konstanz, Konstanz, Germany; ^3^ Computer Science Department, University of Konstanz, Konstanz, Germany; ^4^ Interdisciplinary Centre for the Study of Human Performance (CIPER), Faculdade de Motricidade Humana, University of Lisbon, Cruz Quebrada-Dafundo, Portugal

**Keywords:** attractor method, human cyclic motion, triathlon, running biomechanics, brick run

## Abstract

Running in a triathlon, a so-called brick run, is uniquely influenced by accumulated load from its preceding disciplines. Crucially, however, and irrespective of race type, the demands of a triathlon always exceed the sum of its parts. Triathletes of all levels commonly report subjectively perceived incoordination within the initial stages of the cycle run transition (T2). Although minimizing it, and its influence on running kinematics, can positively impact running and overall triathlon performance, the mechanisms behind the T2 effect remain unclear. In the present study, we assessed the influence of the pre-load exercise mode focusing on the biomechanical perspective. To analyze inertial sensor-based raw data from both legs, the so-called Attractor Method was applied. The latter represents a sensitive approach, allowing to quantify subtle changes of cyclic motions to uncover the transient effect, a potentially detrimental transient phase at the beginning of a run. The purpose was to analyze the impact of a pre-load on the biomechanics of a brick run during a simulated Olympic Distance triathlon (without the swimming section). Therefore, we assessed the influence of pre-load exercise mode on running pattern (*δ*M) and precision (*δ*D), and on the length of the transient effect (t_T_) within a 10 km field-based run in 22 well-trained triathletes. We found that *δ*D, but not *δ*M, differed significantly between an isolated run (I_Run_) and when it was preceded by a 40 km cycle (T_Run_) or an energetically matched run (R_Run_). The average distance ran until overcoming the transient phase (t_T_) was 679 m for T_Run_, 450 m for R_Run_, and 29 4 m for I_Run_. The results demonstrated that especially the first kilometer of a triathlon run is prone to an uncoordinated running sensation, which is also commonly reported by athletes. That is, i) the T2 effect appeared more linked to variability in running style than to running style *per se* ii) run t_T_ distance was influenced by preceding exercise load mode, being greater for a T_Run_ than for the R_Run_ condition, and iii) the Attractor Method seemed to be a potentially promising method of sensitively monitoring T2 adaptation under ecologically valid conditions.

## Introduction

Triathlon involves consecutive swimming, cycling and running over a variety of distances and formats (1, 2). At the elite, but not at the amateur (or so-called age-group) level of competition, the cycle section is draft-legal. These factors in turn affect both the level and the distribution of exercise intensity that the athlete experiences within each individual discipline of a race (3, 4). Crucially, however, and irrespective of race type, the demands of a triathlon always exceed the sum of its parts. Both, triathlon cycling**,** and running are influenced by the demands of their preceding discipline (s), with the most obvious effects of this being manifested up to approximately seven minutes from the bike dismount (5). Over the cycle-run transition (T2), defined as the period from the last kilometer of the cycle section through to the end of the first kilometer of the run, athletes often sense a lack of coordination (5). This may result in altered running kinematics, with adverse consequences for the athletés running performance (6). The overall relative contribution of this triathlon run to race performance generally differs with event distance, within both non-drafting and draft-legal triathlon (7–10). In draft legal triathlon, it has been demonstrated to become increasingly decisive, the better the athletes (11–15). This was shown clearly by the analysis that was carried out by Piacentini et al. (16) of the performance, over the two Olympic cycles from 2009 to 2016, of competitors in the World Triathlon Series (WTS), i.e., the highest level of Sprint and Olympic distance (OD) competition below the Olympic Games. Athletes were divided into 4 groups according to their final race placing (G1: 1^st^–3^rd^ place; G2: 4–8^th^ place; G3: 8–16^th^ place and G4: ≥ 17^th^ place). For females, there were significant differences in the swim and bike segment only between G4 and the other groups, whilst for the run segment each group differed significantly from each other. For males, there were significant differences in swim only between G4 and the other groups, whilst for the running segment each group differed significantly from the others. Essentially, the athletés swimming ability affected how many seconds they exited the swim behind the race leader, and their capability to attain the leading bike pack(s)- within which it was apparently important for overall success that a good runner be positioned (17, 18). Importantly, over all the years and races that were analyzed, both the female and the male winners had, on average, the 2nd run split; the second finisher exhibited, on average, the 4^th^ run split; whilst the third finisher had, on average, the 5^th^ run split -despite there being no particular differences between the first three athletes in their position at the exit from T2. Analysis, over six World Championships and three Olympic Games, of the time lags in (1.5 km/40 km/10 km) OD competition between the first triathlete who started running and the rest of the athletes who arrived in the transition area with the same pack has also confirmed time lost in T2 to be inversely related to performance in males (19). The higher the level and performance density of the race field, the more important a good T2 became. Based on their results, Piacentini et al. (16) consequently suggested that “both for males and females, it is worthwhile to train the actual practice of T2 transitions.’ This advice can also be applied to age-group athletes – for whom prior cycling appeared to have more of an adverse effect on subsequent running than it did in elites (20). However, it is unclear how best to devise such brick workouts, i.e., back-to-back training sessions in multiple exercise modes (2, 5), in an optimal manner, and so ensure a smooth transition from cycling to running. Prior cycling (6, 18, 21) has certainly been shown to elicit changes in neuromuscular (22), physiological (5, 23, 24) and biomechanical (20, 25, 26) parameters, especially during the initial minutes (or transient phase) of the run, although energy availability may also play a role (2, 5, 27). However, and in spite of the amount of research that has been undertaken on this topic to date, it is not yet clear why the phenomenon occurs. One out of various theories (e.g., 26, 27), is that cycling destroys the activity pattern of a subsequent run as a result of differences in the working conditions of the muscles between the two disciplines. Presumably -given that the relevant neuronal and muscular units were pre fatigued and needed time to adapt- the motor program that had been established for cycling could not be instantaneously switched to that of running (6, 28). The problem with elucidating which mechanism(s) underly the T2 response, however, is that it is not easy to examine the effects of the bike-run transition (as compared to control running) in detail. Conventional biomechanical approaches, such as the analysis of step-characteristics (29), ground-contact time (30), or lower limb range of motion (31), often lacked the ability to quantitatively discriminate between subtle running differences because they were focusing only on a part of the content of running motion. Consequently, they ran the risk of disregarding essential gait related information (32, 33).

Nor, although the principle of specificity indicates that brick workouts make sense, has much research to support them been conducted (34–36). While assessment of the ability to run after cycling within a triathlon specific test protocol (37), as well as on its own, is important, neither task has been easy to conduct under ecologically valid conditions (2). Added to that, exactly what constitutes these ecologically valid conditions itself varies with event distance and format. Of the five existing cycle-run transition test protocols that were reviewed by Vleck and Alves (37), for example, three (27, 38, 39) are laboratory- and two (40, 41) are field-based. The former involved ergometer-based cycling and treadmill running – both of which exercise modes differed kinematically from their real world, field based, equivalents (42). Although they appeared to be able to distinguish between neuromuscular adaptors *vs*. non-adaptors to T2; and were sensitive both to differences in T2 adaptation between both genders, between short-distance and long-distance specialists, and between Senior and Junior National Squad athletes, all the laboratory-based tests were and are still somewhat time consuming. Nor may all age-group athletes, as opposed to National Squad level athletes, be able to complete them. As for the two field-based methods, the level to which the specifics of their protocols were appropriate proxies for the demands of actual racing, and/or sensitive to training induced changes in a given athletés adaptation to T2, may vary with athlete level and race format (40, 43). Furthermore, they have, as yet only been used to assess the cardiorespiratory, biochemical, and/or pacing responses to bike run transitions. Despite their potential for applied research with real world implications, wearables have not yet been used to assess the biomechanical response to a bike run transition in the field (44, 45). To put the potential interest of the development of such a system into context, we note that ‘the shorter the race distance and the higher the exercise intensity that is required, the more important a good cycle-to-run transition (T2) is likely to be to the athlete's overall placing” (4). It is not known to what extent fast run starts occur within elite (0.75 km/20 km/5 km) Sprint distance triathlons – although they can account for approximately a quarter of WTS events- but they have been recorded within the (0.3 km/8 km/2 km) Triathlon Mixed Team Relay- which debuted in the recent Tokyo Olympic Games (46). At the elite level the opportunity to evaluate the athletés response to a cycle run transition under race conditions, in conjunction with the ability to provide him/her with speedy feedback on the potential relationship between this and his/her race performance, is becoming increasingly desirable (2, 47).

The Attractor Method, as introduced by Vieten, Sehle and Jensen (32), which regards human motion as affected by stochastic portions, may provide a solution to the problems of sensitivity of the data so obtained, the need for ecological validity of, and the ease providing feedback on adaptation to the cycle-run transition. The outcomes of the method i.e., attractors– which are arrived from the computation of three-dimensional acceleration data from sensors that are attached to the athletés ankles, allow for very precise insights into individual movement behavior of the lower kinematic chain. A special role in research into the triathlon cycle-run transition may possibly be attributed to the so-called “transient effect” (48). The latter described a temporary variation in running characteristics over less than ten minutes after the start of a running session, before the motion stabilizes over time.

The aims of this study were to assess whether the Attractor Method is indeed sensitive to the effects of a (simulated Olympic Distance) cycle-run transition, by using it to quantify the changes in running motion that occurred over an isolated 10 km run (I_Run_) and comparing the data so obtained with those acquired for when the same 10 km (_1–10km_) run was preceded either by an endurance cycle (T_Run_), or by another run (R_Run_) - the energetic load of which was matched to that of the cycling load within T_Run_. The method essentially provided two distinct parameters, how the running changed over, e.g., two time points during a race: δM described changes in the running motion itself and δD reflected the variability (or precision) of the motion. Given that the movement characteristics of cycling differ from that of endurance running, we hypothesized that the magnitude to which both, δM (H1) and δD (H2), within T_Run_ and R_Run_ diverged from their values at equivalent points (_1–10km_) within the isolated run (I_Run_). Thereby, the following hypotheses were formed: H1 = (δM_1–10km_ T_Run_ - I_Run_) > (δM_1–10km_ R_Run_ - I_Run_) for running style as well as H2 = (δD_1–10km_ T_Run_ - I_Run_) > (δD_1–10km_ R_Run_ - I_Run_) for running precision. We also aimed to evaluate the potential use of the duration of the transient effect (t_T_) that occurred prominently over the initial minutes of running exercise (48) as a marker of the extent to which an athlete had adapted to T2. We therefore hypothesized (H3) that t_T_ would be biased by the unfamiliar cycling motion within the preload of, and last longest during the T_Run_. As we would expect the running preload of the R_Run_ to act as a run-specific warm up for the 10km run, we hypothesized that the next longest duration of the transient effect, across our three experimental conditions, would be observed for the I_Run_, leading to the order t_T_: t_Tdistance_ T_Run_ > t_T__distance_ I_Run_ > t_T distance_ R_Run_ for H3.

Lastly, and as a result of conducting the entire study outside the laboratory environment, we aimed to explore the potential of the Attractor Method to translate research into applied practice, by providing rapid real world T2 training and/ or racing related feedback in the field.

## Materials and methods

### Participants

A total of 22 well-trained, but non-elite, athletes (Table 1), 10 of whom were female and 12 of whom were males, were tested over the period June 2021 until October 2021, at the University of University of Konstanz, Konstanz (Germany). In the preparation of the study, the aim was to include a balanced proportion of male and female triathletes. All the study participants were regularly physically active, and none of them was suffering from any injury that could possibly have impeded their performance. They further were actively training and competing in triathlon, having at least three starts in a triathlon race. They agreed that they did not undergo an intensive modification of their individual running technique within the past three months. The study prerequisites were to be aged 18 years or older and be able to run 10 kilometers faster than 50 min (for females)/45 min (for males) and to have a cycling anaerobic threshold (AT) of at least 2.5 W/kg (for female)/3.5 W/kg (for male). The above performance-relevant parameters were ensured in advance using remote performance diagnostics (PPD-Cycling and PPD-Running, INSCYD GmbH, Switzerland, see https://inscyd.com/functions/power-performance-decoder). All participants provided their written informed consent. The study was approved by the local University of Konstanz Ethics Committee (Ref. No.: IRB21KN008-01w).

**Table 1:** Participant overview (F = female, M = Male, All = both, provided as mean (SD)).

	N	Age (year)	Height (cm)	Mass (kg)	Triathlon experience (years)	$\dot{V}$O_2_max cycling (ml/min/kg)	$\dot{V}$O_2_max running (ml/min/kg)	Speed brick run (% of anaerobic threshold)
F	10	31 (9.2)	168 (6.4)	60.2 (6.7)	5 (4.7)	49.6 (3.6)	49.3 (4.1)	96.4 (6.3)
M	12	28 (6.1)	179 (5.1)	72.3 (5.0)	7 (3.8)	59.7 (7.0)	61.1 (4.2)	98.1 (3.9)
All	22	29 (7.8)	174 (8.1)	66.8 (8.4)	6 (5.6)	55.4 (7.4)	56.0 (6.9)	97.3 (5.1)

### Equipment & test setting

#### Sensor technology

To collect the necessary raw data, two inertial sensors were used (SpoSens 2.0, Wille Engineering, Germany). The sensors had a size of 77.5 × 37 × 34.5 mm and weighed 45 g each. They functioned as a triaxial accelerometer with up to 400 G (16 G was used), a triaxial Gyroscope with up to 2000°s (maximum was used) and a magnetometer measuring with up to 16 Gauss (maximum was used). The possible sampling rate could be set up to 1.000 Hz (250 Hz was used) and they were constructed as a micro-electro-mechanical system (MEMS). Further, the sensors were equipped with GNSS (max. 5 Hz) technology. They collected and saved the running motion data in three dimensions (x, y, z) on an internal storage device (8192 GB). The sensors were attached to both ankles above each lateral malleolus by a hook-and-loop fastener. All data were received and stored on a GARMIN Forerunner 945 (GARMIN, Schaffhausen, Switzerland) during the entire test session. Earlobe blood samples (each of 10 µl) were analyzed for lactate concentration using a stationary laboratory device (HITADO Super GL compact). Body weight, body fat percentage and active muscle mass (required for the INSCYD analysis) were measured with a Tanita BC-545N body analysis scale (Tanita Europe B.V., Stuttgart, Germany).

#### Run course and bike setting

**Figure 1:**
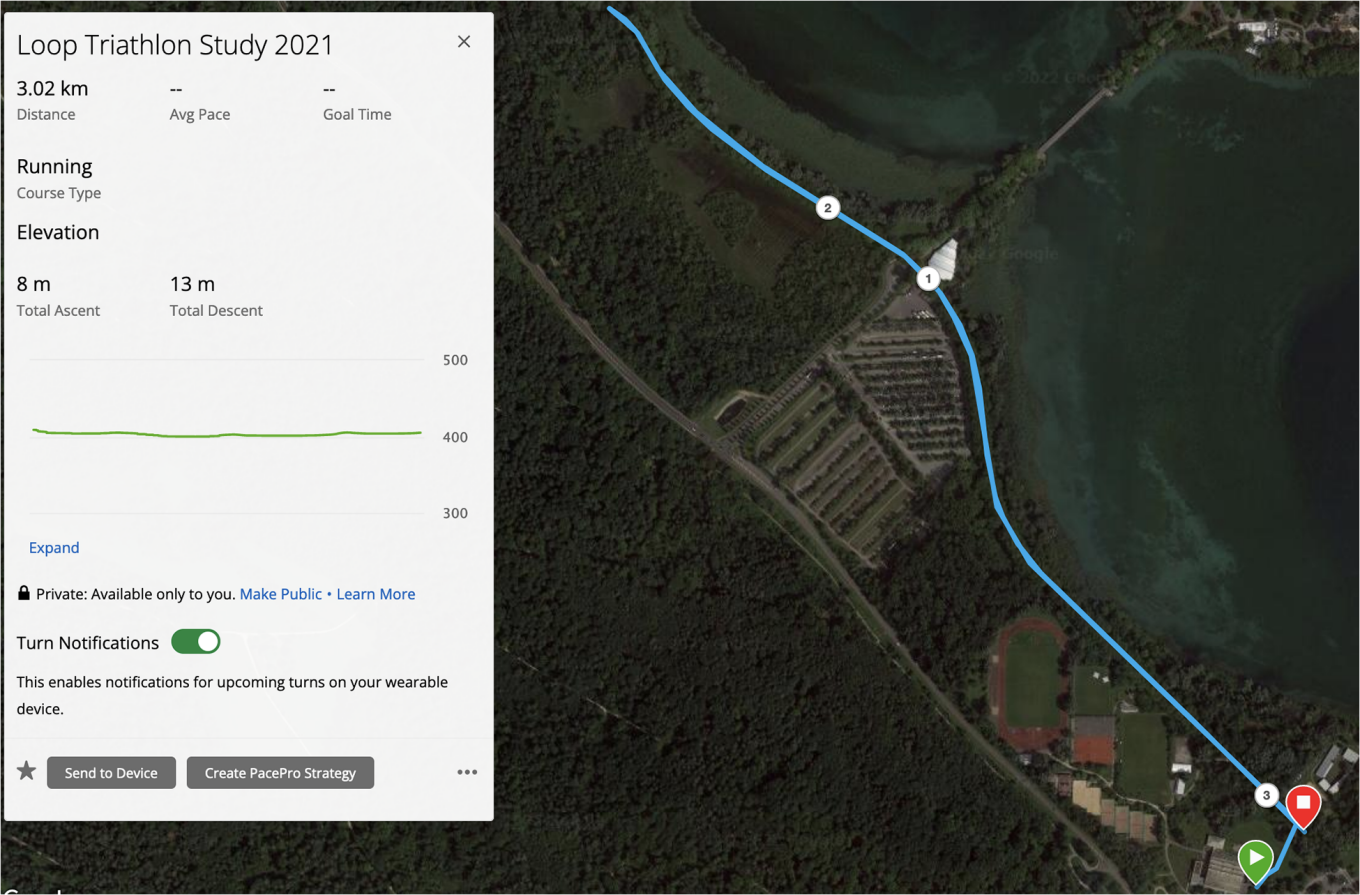
Running course map (extracted from Garmin Connect App, GARMIN, Switzerland).

The runs were performed outside at the Graf Lennart Bernadotte Allee in Konstanz (Germany). This is a cycling path, between the sports facilities of the University Konstanz and the Island of Mainau, that is generally closed to traffic (see Figure 1).

**Figure 2:**
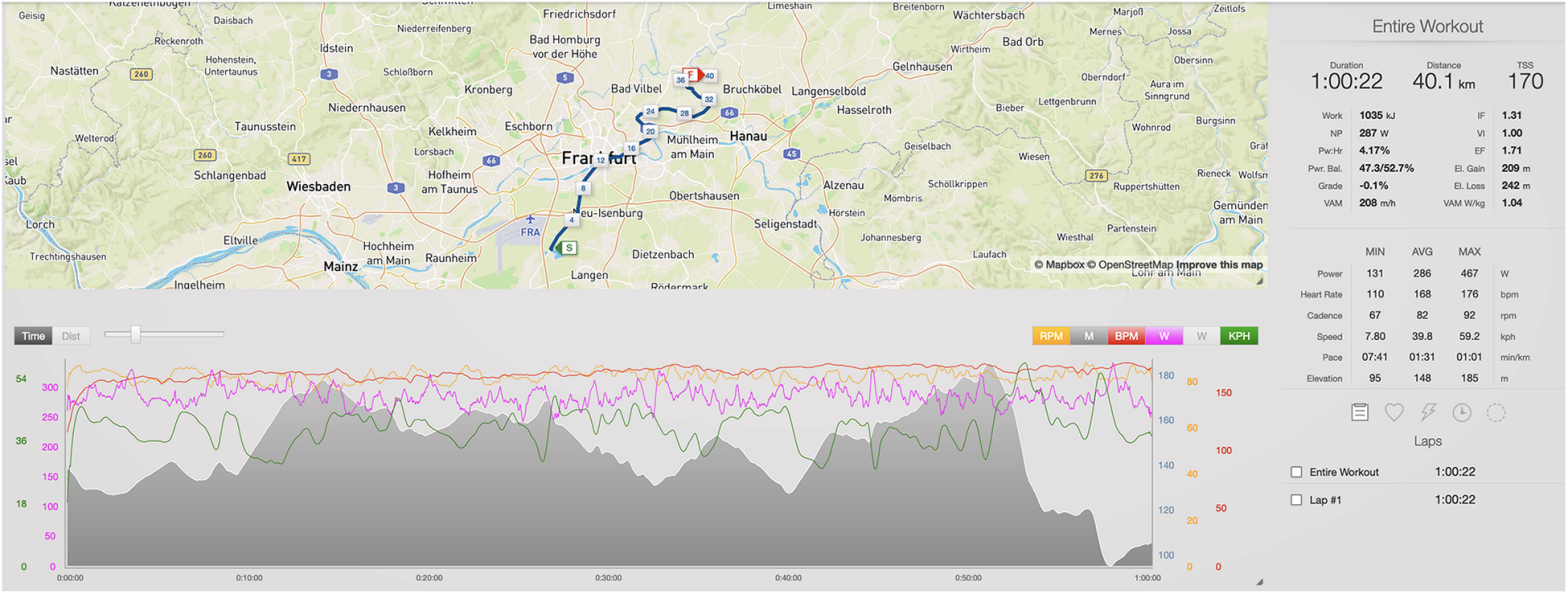
Cycling route (extracted from TrainingsPeaks, Peaksware, United States of America).

This three-kilometer loop was completely flat and had to be ran 3.5 times to achieve the 10 km run distance equivalent to that which occurs within an Olympic distance triathlon. The running preload, which had an individually calculated length and intensity, was also carried out over this route. For the triathlon test all participants had to undergo a 40 kilometer cycling time trial on an indoor smart trainer (Tacx NEO 2T, Tacx, Wassenaar, Netherlands) using their own road bike. As the route to be cycled we chose the first 40 kilometer of the IRONMAN Frankfurt (see Figure 2) which represented an appropriate mixture of flat and hilly sections (accumulated altitude meters 208 m). The simulation was undertaken with the indoor cycling reality app Rouvy (VirtualTraining s.r.o., Vimperk, Czech Republic).

### Experimental protocol and data measurement

#### Determination of performance parameter

The study was conducted as a cross-over study with three conditions: Triathlon Run (T_Run_), Run-Run (R_Run_) and an Isolated Run (I_Run_) separated by at least 5 days. To set the right power for the cycling simulation and the running pace for the preload of the Run-Run condition each athlete had to undergo a performance diagnostic in both disciplines in advance. For both analyses the Power Performance Decoder (PPD) of INSCYD (Switzerland, see https://inscyd.com/functions/power-performance-decoder) was used. This performance tool allowed remote performance testing, so that all participants could gather their data on their own after receiving prior instructions *via* video call. Finally, based on these outcomes, we had access to the calculated (maximum) oxygen uptake ($\dot{V}$O_2_max) and the (anaerobic) threshold power/pace, needed for further use. Additional training status related feedback parameters that the tool provided, such as the lactate building rate ($\dot{V}$_La_max), substrate consumption or derived training zones were offered to the participants after they competed the entire test scenario.

#### The triathlon test (T_Run_)

**Figure 3:**
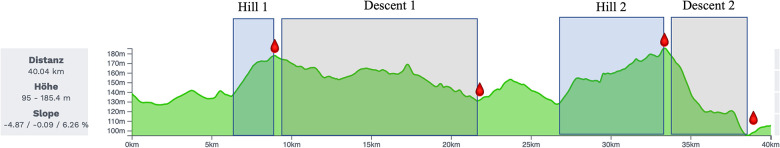
Cycling course profile with hills and descents.

The basic initial test session was always the triathlon condition (T_Run_), as this was needed to get reference values for both other experimental conditions. The T_Run_ consisted of a 40-kilometer time trial (Figures 2,3) on a smart trainer at 90%–95% of the individually determined threshold power, i.e., average power within the cycle section of an Olympic distance triathlon (see Table 2 in 3,4). Due to the often draft-legal racing formats, the pacing (in OD races) is rather characterized by an intermittent character (49). To represent this, athletes were instructed to ride significantly above the given power range on two climbs (see Figure 3, blue area), followed by sections below this range on the subsequent descents (grey area). The entire ride was carried out in the handlebar position with no use of aerobars being allowed (50).

**Table 2:** Distance over which transient effects (TE) were observed in meters. The cases who showed no transient effect are labelled as “no TE”. f = female; m = Male subject.

Subject	I_Run_ [m]	T_Run_ [m]	R_Run_ [m]	Subject	I_Run_ [m]	T_Run_ [m]	R_Run_ [m]
1 (m)	380	200	232	12 (m)	no TE	1600	940
2 (f)	60	20	no TE	13 (f)	180	no TE	no TE
3 (m)	180	550	no TE	14 (f)	no TE	1400	no TE
4 (m)	no TE	338	no TE	15 (f)	228	no TE	no TE
5 (m)	no TE	238	18	16 (f)	no TE	690	482
6 (m)	20	1180	no TE	17 (f)	no TE	462	354
7 (m)	no TE	160	no TE	18 (f)	no TE	28	760
8 (m)	no TE	no TE	280	19 (m)	no TE	no TE	no TE
9 (m)	no TE	250	600	20 (f)	no TE	720	no TE
10 (m)	no TE	540	no TE	21 (f)	1012	610	no TE
11 (m)	no TE	2000	380	22 (f)	no TE	1234	no TE

The cycling part was followed by a 10km all-out outdoor run (Figure 1). The pacing could be chosen individually. The change between both disciplines had to be done as fast and triathlon specific as possible. The transition time was noted. Beforehand the athletes were equipped with several sensors (see above) placed in such a way, that they did not interfere with the sport-specific execution of running and cycling. All the listed devices recorded the corresponding data during the entire T_Run_. During the cycling section lactate samples were collected form the earlobe after both hills and descents (Figure 3) to check if the athletes had followed instructions to increase and lower their intensity. Further samples were taken immediately after the bike session and right before the start of the run. Finally, blood lactate concentrations were determined immediately after the 10 kilometer run and at a further 1 min, 3 min, 5 min and 7 min post-finish. The participants were instructed following an appropriate carbohydrate diet before and during the test, as well as regarding an adequate training load to present themselves for testing in a fully recovered state, with replenished glycogen stores. The runners were accompanied throughout the entire trial by a cycling member of the scientific team in order both to provide them with nutrition (when requested) and ensure their safety.

#### The run-run test (R_Run_)

The R_Run_ condition matched the T_Run_ but instead of the cycling preload the athletes had to run an individually tailored running session. This multiple-step calculation was carried out by metabolic matching of cycling and running loads, based on the performance diagnostic data (INSCYD PPD, see https://inscyd.com/functions/power-performance-decoder), as follows:

(1) Active muscle mass for cycling (60% of muscle mass) and running (70% of muscle mass) were determined based on individual weight (BW), body fat percentage and muscle mass.
(2) The aerobic capacities ($\dot{V}$O_2max_) of both disciplines were multiplied by BW and divided by the active muscle mass for (1) to get the absolute amount of oxygen used by the active muscle mass in millilitre per minute.
(3) The average power output (in watts) during the T_Run_ cycling section could be attributed to an individual oxygen consumption. The latter was taken from the performance diagnostics and calculated as in (2) only for the active muscle mass.
(4) The ratio between the active muscle mass used during the cycling session (3) and the individual $\dot{V}$O_2max_ (2) resulted in the proportional utilization of the $\dot{V}$O_2max_.
(5) This ratio was transferred to the calculation of the running effort. On this basis, steps (3) and (4) were performed backwards, so that ultimately an oxygen uptake for the running section was obtained that was metabolically equivalent to the cycling load.
(6) The calculated oxygen consumption was then assigned to an appropriate running pace using the data from the performance diagnostics.

As a last step, the running duration was adjusted due to the significantly higher orthopaedic stress during running (weight-bearing *vs.* non-weight-bearing exercise). This weight-bearing-factor based on the observations of Munro, Miller & Fuglevand (51). The latter provided vertical ground reaction forces (GRF) as an Impact maximum (See Table 3 in 51, Table 3) relative to BW differentiated according to the individual running speed. To determine the duration of the preload run, the duration (in full minutes) of the cycling section of the T_Run_ condition, was divided by the running pace-related weight-bearing-factor.

The duration between the preload and the brick run was adjusted to the time the athlete spent in the T_Run_ condition. The brick run (R_Run_) was paced by a GARMIN sports watch each kilometer, according to the kilometer splits that the athlete had run during the baseline test (T_Run_) ± two seconds. Again, the athletes were supported by a cyclist and the lactate samples as well as the data collection were undertaken similarly to as in the T_Run_.

#### The isolated run test (I_Run_)

The Isolated run (I_Run_) was paced by a GARMIN sports watch each kilometer, according to the kilometer splits that the athlete had had run during the baseline test (T_Run_). All the study participants were allowed to warm up for 10 min before I_Run_, although none of them did so. The athletes were supported by a cyclist and the lactate samples as well as the data collection were undertaken similarly to the T_Run_. The order of I_Run_ and R_Run_ was set randomly.

### Data analyses

To detect subtle changes in the running behavior after the preloads, the Attractor Method was used to create an individual attractor from each kilometer, which then served as the basis for further processing steps. We evaluated whether there was a statistical difference between the T_Run_ and the R_Run_ conditions as compared to the basic run (I_Run)_, and if the differences in transient time that we expected between conditions could be seen. Furthermore, conventional running analysis parameters, like stride frequency and ground contact time were statistically examined.

#### Attractor parameter

Based on three-dimensional acceleration data, the Attractor Method (see 33, *p*.3 for the complete mathematical derivation) allowed the determination of two main parameters describing changes in human cyclic motion. As a basis served the calculation of an attractor $\vec{A}$ representing each measuring interval, like one kilometer of running, (equation 1) and its fluctuations *D* (equation 2).

(1) $${\vec{A}}_{a,C}(\tau_{j})=\frac{1}{n}\overset{n}{\underset{i=1}{\sum }}{\vec{a}}_{a,C}(i\cdot \tau_{j})+\frac{1}{n}\overset{n}{\underset{i=1}{\sum }}{\vec{b}}_{a,C}(t=i\cdot \tau_{j})\approx \frac{1}{n}\overset{n}{\underset{i=1}{\sum }}{\vec{a}}_{a,C}(i\cdot \tau_{j})$$

(2) $$D_{a,C}(\tau_{j})=\sqrt{\frac{1}{n-1}\;\overset{n}{\underset{i=1}{\sum }}[{\vec{A}}_{a,C}(\tau_{j})-\;{\vec{a}}_{a,C}(i\cdot \tau_{j}){]}^{2}}$$

with *t* being the time, *a* the right or left foot ankle (where sensors were attached) and *C* represented two different time/distance intervals of the compared run.

Subsequently, the two parameters, one describing changes in the motion itself (δM, equation 3) and further, the variability of the motion (δD, equation 4) can be calculated.

(3) $$\delta M\;=\frac{1}{v}\sqrt{\overset{3}{\underset{i=1}{\sum }}\left[\left\langle {\left(A_{r,B,x_{i}}-A_{r,E,x_{i}}\right)}^{2}\rangle +\;\langle {\left(A_{l,B,x_{i}}-A_{l,E,x_{i}}\right)}^{2}\;\right\rangle \right]}$$

with *v* being the running speed in m/s, *r* and *l* stood for right or left foot ankle (where sensors were attached) and *B = begin* and *E = end* represented two different time/distance intervals of the compared run. < *…*> meant the average of the included expression.

δM described the velocity normalized difference between two attractors (30), allowing to quantify the two time points or the running conditions of this study, regarding changes in the individual running motion.

(4) $$\delta D=\sqrt{\langle {\left(D_{r,B}-D_{r,E}\right)}^{2}\rangle +\langle {\left(D_{l,B}-D_{l,E}\right)}^{2}}\rangle$$

δD corresponded to the difference between the variability around each of the two attractors and was therefore a proxy measure for the precision of a movement. Its calculation was based on the absolute *D* (equation 2) of each attractor, which described the average deviation of single gait cycles from the attractor. For the calculation of both, the research group provided an open access application (available online http://www.uni-konstanz.de/FuF/SportWiss/vieten/CyclicMove/). In the present study, based on previous work (for an overview see 34), δM and δD values equal to or below 5 m/s^2^ represented a very high similarity of the compared running motion and the movement precision respectively.

#### Attractor analysis

All runs were split into single data sets, each of which represented one kilometer within each of the three conditions (T_Run,_ I_Run_, R_Run_), on the basis of the athletés recorded GPS signals. This allowed for fluctuations in pacing to be considered in the subsequent evaluation. In additional to being the normal cycle and run unit that has been considered within triathlon cycle and run pacing research (17, 18) a one-kilometer-separation resulted in average interval lengths of four to five minutes (per kilometer). This was an appropriate duration for analyses of running motion to have sufficient cycles for a meaningful attractor calculation. The δM and δD values derived from comparisons of the single kilometers 1–10 between the control I_Run_ session, and each of the two brick runs: T_Run_ and R_Run_, served as the basis for further statistical calculations.

#### Transient effect analysis

In a second analysis process the running data from each condition were split into fifty 200 m sections, so as to obtain a more precise insight into when the transient effect of a running session ended, and the athletes finally adapted a smooth running motion. To perform the transient analysis, the procedure that has been extensively described in Weich et al. (48) as “I. Delta M (δM)” was followed, using a slightly modified equation for δM:

(5) $$\delta M_{transient}=\frac{1}{v}\cdot \langle T_{∥}\cdot \left[e^{\frac{-t}{t_{T}}}-e^{\frac{-t_{E}}{t_{T}}}\right]+\;a_{0}\cdot \left\{\sqrt{\frac{\left(t_{E}-t\right)}{t_{E}}}+a_{1}\cdot sin\left(a_{2}\cdot 2\pi \frac{\left(t_{E}-t\right)}{t_{E}}\right)\right\}\rangle$$

with the given constants $T_{∥},t_{T},a_{0},a_{1},a_{2}$, which are derived from a curve fitting application of all measurements (CurveExpert Professional (version 2.6.5, Hyams Development)), using the Levenberg-Marquardt algorithm. The constants $a_{0},a_{1},a_{2}$ represented a morphing process whereas $T_{∥}\;\mathrm{and}\;t_{T}\;$were based on the transient oscillations at the onset of a movement (see 52 for a full description of all components of cyclic human motion). $t_{T}$ quantified the time until the oscillation decreased to $e^{-1}$ of its original starting value *T*.

In the final result, the durations of the transient effect ($t_{T}$) were given as a distance in meters for each individual run of each person. Runs which did not show a transient effect are reported (Table 2) but were not included in the average distance calculation.

### Statistical analysis

The traditional stride data, stride frequency and ground contact time, were analyzed using a two-way repeated measures ANOVA (SPSS, IBM Version 28) with one factor being the distance in ten-kilometer levels and the second the three running conditions. The stride lengths were not analyzed because they are inversely related to stride frequency. The attractor-based data were evaluated with pairwise Student's t-tests - comparing the δM and δD outcomes of both comparisons (I_Run_ vs. T_Run_ and I_Run_ vs. R_Run_) within each kilometer. To check whether the athletes' pacing targets, based on the initial brick run, were met, running speeds (as a percentage of the average time for each running condition) were compared, on a kilometer-by-kilometer basis, between the three experimental conditions using a repeated measures ANOVA (SPSS, IBM Version 28). This included looking at potential gender differences. The significance level was set at *p* < 0.05.

## Results

### Conventional parameter and pacing

**Figure 4:**
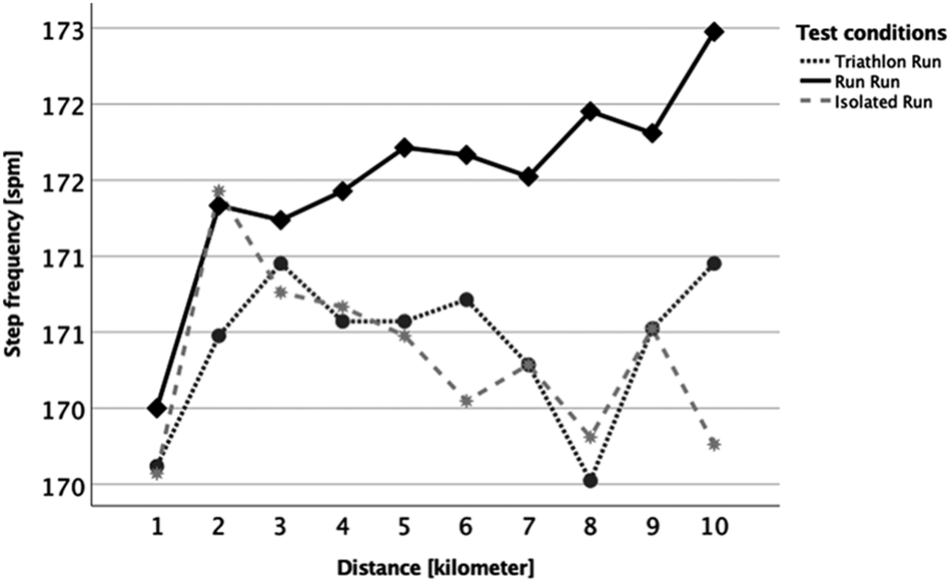
Kilometer-separated development of step frequency between the three running conditions (Triathlon Run = dark grey dotted line; Run Run = black solid line; Isolated Run = grey dashed line).

**Figure 5:**
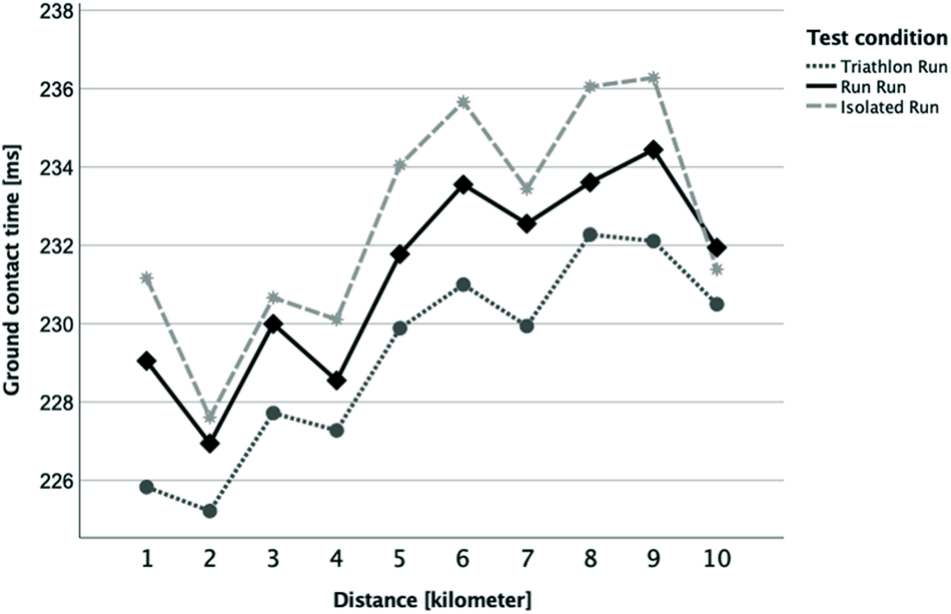
Kilometer-separated development of ground contact time between the three running conditions (Triathlon Run = dark grey dotted line; Run Run = black solid line; Isolated Run = grey dashed line).

When examining the traditional parameter of stride frequency (Figure 4) there were no statistically significant differences (*p* > 0.05 between all kilometers, conditions, and interaction) until kilometer 5. For kilometers 6 (*p* = 0.040), 8 (*p* = 0.002) and 10 (*p* = 0.003) the R_Run_ was significantly different from the I_Run_ condition (but not from the T_Run_). One noticeable aspect was that the first kilometer initially started with a low cadence but then increased over the second and third kilometer in all running categories, and that the stride frequency increased steadily over the entire distance in the R_Run_ condition. Another traditional parameter was the ground contact time (GCT, Figure 5), where the conditions and the interaction (distance*condition) showed no significant difference (*p* > 0.05). Although the first kilometer in all conditions initially started with a somewhat elevated GCT before the second kilometer always indicated the lowest GCT. For the within-factor distance there was an overall significant difference with post-hoc tests (Bonferroni correction) revealing kilometer 2 to be significantly different from kilometers 6, 8 and 9.

In terms of pacing, we typically observed a u-shaped pattern, i.e., a higher initial pace above 100% of the later average pace of the whole run, followed by a gradual decrease in running speed to about 98% of the average pace up to kilometer 8, before the last two kilometers were covered significantly faster (at about 100% of the average pace). This was true for both genders. There was no significant difference in running speed between the running categories (I_Run_, T_Run_, R_Run_) over any kilometer, indicating a very good compliance with the pace prescriptions and allowing a reliable comparability for the attractor-based motion analyses. The average transition time between cycling and running (T_Run_) was 2.6 (SD 1.2) minutes. The runners were instructed to replicate their transition time from the first test (T_Run_) during the R_Run_.

### Attractor-based motion analyses

**Figure 6:**
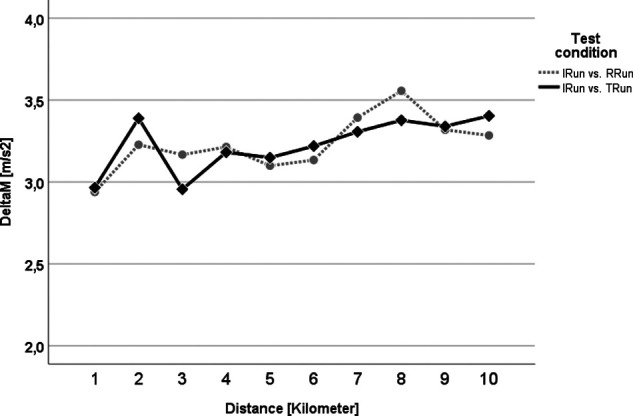
δM analysis for the control run vs. the T_Run_ (black solid line with rhombus) and the R_Run_ (grey dotted line with circle). No statistical difference can be seen.

In the pairwise δM comparison for each kilometer between the I_Run_ and both brick conditions (T_Run,_ R_Run_) no statistically significant differences (at the *p* > 0.05 level) could be seen (Figure 6). Although within both conditions, T_Run_ and R_Run_, δM increased slightly over the course of the runs, its absolute size remained well below the cut-off of δM = 5 m/s^2^ i.e., both conditions were very similar in motion terms.

**Figure 7:**
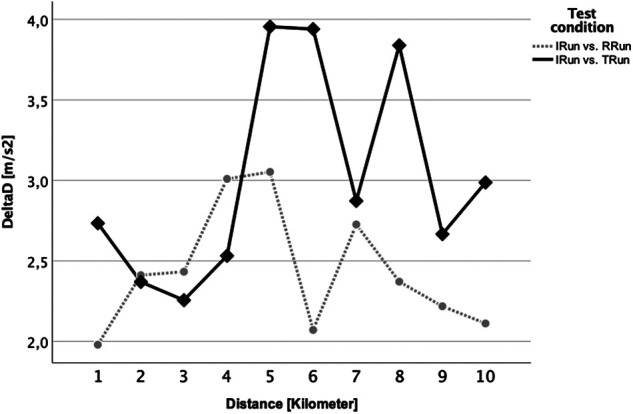
δD analysis for the control run vs. the T_Run_ (black solid line with rhombus) and the R_Run_ (grey dotted line with circle). Kilometer 1 and 10 indicate a significant difference between both conditions.

For δD, reflecting the difference in variation of the running motion, we saw equally generally low δD scores clearly below 5 m/s^2^ (Figure 7). Here, the T_Run_ usually showed higher values and thus a higher range of variation, for kilometer 1 (*p* = 0.026) and 10 (*p* = 0.011) being significantly different to the same kilometer within the R_Run_.

### Transient effect

**Figure 8:**
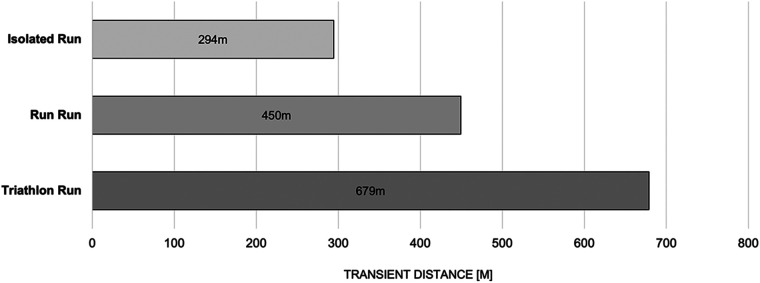
Overview of the different distances that were covered to finish the transient process at the initial stage of a run. Only sessions with a measured transient effect are included in the plot.

Table 2 and Figure 8 present an overview of all athletes' occurrences of the transient effect, together with the distance over which it was in effect. During both running conditions, the I_Run_ and the R_Run_, athletes experienced a transient phase in only 32% and 41% of cases, respectively, whereas the triathlon preload caused the initial effect in 82% of the group. It was evident that both brick conditions took the longest way for the runners to find their running rhythm: 450 m (min. 18 m, max. 940 m), a bit more than one lap on a usual track in a stadium, during the R_Run_ and almost 700 m (min. 20 m, max. 2000 m) during the T_Run_. For the I_Run_, on the other hand, the few participants who showed any transient effect at all, did not even need 300 m (min. 20 m, max. 1012 m) to get into their running rhythm.

## Discussion and practical implications

Our first aim was to quantify the running behavior of triathletes with and without preloads. We assessed multiple biomechanical and pacing related parameters between an isolated run (I_Run_) and two brick runs, one involving a previous run (R_Run_) and the other, as per within a triathlon, involving a prior bike load (T_Run_). In this way, the uncoordinative feeling of triathletes, which many experience during the early phase of their run in a competition, was objectively quantified under field conditions. Our intention was further to improve on the methodology of our initial triathlon paper (21). That meant both, an improved evaluation algorithm of the Attractor Method, and an improved study design i.e., one that used the additional Run-Run condition to test whether cycling *per se* impairs running or preloads in general. In the aforementioned study, the authors noticed that the athletes' running behavior was quite messy within the first five minutes after the run start, until the athletes finally found their running rhythm. That this effect applied, even independently of the preload, was also confirmed by the results presented here. Moreover, it was not surprising that this transient effect, which was described by Weich, Vieten & Jensen (48) lasted longest after cycling (i.e, 679m, on average, in this study, confirming the t_T_: t_Tdistance_ T_Run_ > t_T__distance_ I_Run_ section of our initial hypothesis H3). The explanation for this phenomenon is likely the different motion pattern of cycling compared to running (28, 53). Particularly remarkable compared to our work from 2020 (48) was the finding that exceptionally high numbers of athletes (indeed, almost 70%) did not exhibit a transient effect in their I_Run_ at all. A plausible reason for this, as already described back in the 2020 paper on a case wise basis, could be the above average experience and performance level of all the participants in the present study (Table 1). Consequently, our results contradicted our expectations (and the t_T__distance_ I_Run_ > t_T distance_ R_Run_ section of our hypothesis H3) to the extent that the I_Run_ not only presented quite few transient cases, but also the shortest duration of all three conditions (294 m). Thus, it seemed that preloads always caused a higher transient duration compared to a stand-alone run started from a resting state (and that t_T_: t_Tdistance_ T_Run_ > t_T distance_ R_Run _> t_T__distance_ I_Run_). This was even true when the preload discipline corresponded to the subsequent exercise mode (R_Run_), although the preliminary runs in this study had always been performed at a more moderate pace. That is, the data that were obtained by this study clearly confirmed, yet again, that a basic transient effect seems to be present in cyclic movements such as running. This effect was prolonged temporarily by a preceding load and was further intensified if said preload involved a different exercise mode, such as cycling. This finding should also encourage research in other (cyclic) sports, especially those with alternating disciplines (duathlon, biathlon, pentathlon etc.), to include the transient effect in their test designs and access its applications.

Furthermore, the runs were also compared between each other using the Attractor Method. Pairwise comparisons of each brick run with the session without a preload were made to highlight how much they differed in running pattern and variability over the entire distance. The statistical analysis showed that for δM, contrary to expectations, i.e., the possible change in the running style itself, there was no significant difference at any time (Figure 6). That attractors are highly individual and stable has already been shown in previous studies for walking (54, 55) and also for treadmill running (56) - which should have reduced the complexity of the movement, especially due to the steady running speed and the even ground of the treadmill belt. Although the δM-related hypothesis of the current study (our hypotheses H1) must be rejected, the results of previous works on the attractor properties appeared to be confirmed even for more complex test designs as applied here. This means that even, or especially, in very trained athletes, that the running style itself remained very stable and other factors must be considered for the feeling of uncoordinated running and the accompanied transient effect as reported above. One of the possible explanations might be an increased variability of the movement, thus an increased variation (δD) within the gait cycles which are summarized in one attractor (32). This parameter was examined in the same way as δM before, however, it revealed a significant difference for the first and the last kilometer (Figure 7). This difference might be attributed to the R_Run_, especially within the first and last kilometer, possessing a higher movement precision as a result of its lower δD value. Accordingly, our initial hypothesis (H2) concerning the motion variability (δD), at least partially, can be confirmed. It was reasonable to assume that for the discrepancy between R_Run_ and T_Run_ at the beginning (kilometer 1) was caused by the transient effect described above, which was probably driven by the variability of the running motion. As noted in previous studies (21, 29, 57), cycling itself appeared to have a very disruptive effect on the subsequent run. This study showed, for the first time, that this most likely influenced the precision and less of running motion itself. This observation should therefore be more closely examined in follow-up studies, in order to be able to derive major practical consequences. That the tactics how to run the first race kilometer could be of high importance has already been shown by works on pacing (58, 59) as well as the contribution of the individual performances to the overall performance in triathlon (17, 18, 60). Changes in running behavior were also commonly seen in the last few meters of a triathlon, as indicated by the statistically relevant difference in δD during the last kilometer, also in the present paper (Figure 7, kilometer 10). Further, analyses of pacing strategies showed, at least with respect to the running speed, a u-shaped development, which reveals a re-increase of the pace towards the end (16, 18). The results of this study suggested that the cycling preload affects the 10km run substantially more than a running preload does. This was furthermore supported by the fact that on the second half of the run, i.e., from kilometer 5, the variability of the running behavior in the triathlon run was permanently higher (although only significantly different on the last kilometer; see Figure 7). In general, it can also be stated that the absolute δM and δD values in this study could be classified as very low (< 5 m/s^2^), which speaks for an extremely similar running motion. This underlined once again the stability of the individual attractors. Furthermore, it should be highlighted that the sensitivity of the Attractor Method to analyze human cyclic motion had been demonstrated by the fact that conventional parameters, such as stride frequency/-length or ground contact time, were unable to represent these findings (Figures 4 and 5).

One limitation to be mentioned here is that the data that we presented were derived from sample of well- to very well-trained endurance athletes. The results are therefore not applicable to untrained or professional athletes without further investigation. The calculation of attractors was mathematically based on mean values of many data points. In this study, the algorithm was applied per kilometer, which lasted about five minutes on average. This corresponded to an average step frequency of 170 steps per minute, 85 complete step cycles per minute and thus 425 cycles per kilometer. This means that if only a few cycles are unrhythmic or distinctive, they will disappear with the averaging process. In future studies, it might be methodologically more appropriate to look at smaller distances, such as 400m (= approx. 2 min and 170 cycles), to be more selective. On the other hand, as seen within kilometers 1 and 10 for δD, if significant differences show up due to the methodology used here, one can assume that they are so severe that they occur for the majority of the entire kilometer.

We already targeted to realize an evenly distributed sample concerning male and female participants. The latter will allow us to evaluate the important difference between the two in a separate study in the future.

Future work should moreover focus on an application of the methodology providing (live) feedback already during the running session. The research group has already developed a beta version of the software that allows live data communication including analyses results within a marginal time delay < 40 ms. A key and sport-practically relevant future objective must be to find out whether the impairment during the brick runs that has been described in this paper has a negative impact on triathlon performance. If this is the case, the Attractor Method may in itself present a promising solution to determine which training and competition strategies can best be used to reduce bike-run related deficits.

## Summary and conclusions

This triathlon transition study used a hands-on methodology and applied tool, based on attractors, to analyze the motion pattern and variation of running after an endurance sports related preload, in the field. While we could not support our first hypothesis (H1), as we did not find (δM_1–10km_ T_Run_ - I_Run_) > (δM_1–10km_ R_Run_ - I_Run_), we did observe that (δD_1–10km_ T_Run_ - I_Run_) > (δD_1–10km_ R_Run_ - I_Run_), confirming the second hypothesis (H2), at least for the first and last kilometer. Moreover, rather than it being the case that t_T_: t_Tdistance_ T_Run_ > t_T__distance_ I_Run_ > t_T distance_ R_Run_, as we had initially hypothesized (H3), we found t_Tdistance_ T_Run_ > t_T distance_ R_Run _> t_T__distance_ I_Run_. The data that we obtained demonstrated that especially the first kilometer of a triathlon run is prone to running in an uncoordinated manner, which has been commonly reported by athletes. The results indicated that the cause of this T2 effect is probably linked not so much to running style itself, but, rather, to variability in it. In a sport like triathlon, which is always striving for marginal gains, this finding could open another perspective both to examine and improve the final discipline, and to be able to do so under ecologically valid conditions. “At minimum, easing (the cycle-run) transition can bring added comfort to the athlete, and at maximum it could mean the (…) difference between victory and defeat” (34).

## Data Availability

The raw data supporting the conclusions of this article will be made available by the authors, without undue reservation.

## References

[1] Bentley DJ Millet GP Vleck VE McNaughton LR. Specific aspects of contemporary triathlon: implications for physiological analysis and performance. Sports Med. (2002) 32:345–59. 10.2165/00007256-200232060-00001 11980499

[2] Walsh JA. The rise of elite short-course triathlon Re-emphasises the necessity to transition efficiently from cycling to running. Sports. (2019) 7:99. 10.3390/sports7050099 31035687 PMC6571801

[3] Aoyagi A Ishikura K Nabekura Y. Exercise intensity during Olympic-distance triathlon in well-trained age-group athletes: an observational study. Sports. (2021) 9:18. 10.3390/sports9020018 33494505 PMC7912546

[4] Vleck V Millet GP Alves FB. The impact of triathlon training and racing on Athletes’ general health. Sports Med. (2014) 44:1659–92. 10.1007/s40279-014-0244-0 25292108 PMC7099231

[5] Millet GP Vleck V. Physiological and biomechanical adaptations to the cycle to run transition in Olympic triathlon: review and practical recommendations for training. Br J Sports Med. (2000) 34:384–90. 10.1136/bjsm.34.5.384 11049151 PMC1756235

[6] Fraeulin L Maurer-Grubinger C Holzgreve F Groneberg DA Ohlendorf D. Comparison of joint kinematics in transition running and isolated running in elite triathletes in overground conditions. Sensors. (2021) 21:4869. 10.3390/s21144869 34300608 PMC8309736

[7] Figueiredo P Marques EA Lepers R. Changes in contributions of swimming, cycling, and running performances on overall triathlon performance over a 26-year period. J Strength and Cond Res. (2016) 30:2406–15. 10.1519/JSC.0000000000001335 26808853

[8] Horne MJ. The relationship of race discipline with overall performance in sprint and standard distance triathlon age-group world championships. Int J Sports Sci & Coach. (2017) 12:814–22. 10.1177/1747954117738878

[9] Scorcine C. Contribution of swimming, cycling and running in the final performance in different distances of triathlon races. MOJSM. (2017) 1:125–8. 10.15406/mojsm.2017.01.00027

[10] Sousa CV Aguiar S Olher RR Cunha R Nikolaidis PT Villiger E Rosemann T Knechtle B. What is the best discipline to predict overall triathlon performance? An analysis of sprint, Olympic, ironman® 70.3, and ironman® 140.6. Front Physiol. (2021) 12:654552. 10.3389/fphys.2021.654552 34025447 PMC8131838

[11] Fernández-Revelles AB Ramírez-Granizo I Castro-Sánchez M Padial-Ruz R. Men's triathlon correlation between stages and final result in the London 2012 Olympic games. Journal of human sport and exercise - 2018 - spring conferences of sports science. Universidad de Alicante (2018) 10.14198/jhse.2018.13.Proc2.35

[12] Fröhlich M Klein M Pieter A Emrich E Gießing J. Consequences of the three disciplines on the overall result in Olympic-distance triathlon. International Journal of Sports Science and Engineering. 2(4):204–10.

[13] Hoffmann M Moeller T Seidel I Stein T. Predicting elite triathlon performance: a comparison of multiple regressions and artificial neural networks. Int J Comp Sci in Sport. (2017) 16:101–16. 10.1515/ijcss-2017-0009

[14] Olaya J Fernández-Sáez J Østerlie O Ferriz-Valero A. Contribution of segments to overall result in elite triathletes: sprint distance. IJERPH. (2021) 18:8422. 10.3390/ijerph18168422 34444171 PMC8394650

[15] Fröhlich M Balter J Pieter A Schwarz M Emrich E. Model-theoretic optimization approach to triathlon performance under comparative static conditions – results based on the Olympic games 2012. Int J Kinesiol & Sports Sci. (2013) 1:9–14. 10.7575/aiac.ijkss.v.1n.3p.9

[16] Piacentini M Bianchini L Minganti C Sias M Di Castro A Vleck V. Is the bike segment of modern Olympic triathlon more a transition towards running in males than it is in females? Sports. (2019) 7:76. 10.3390/sports7040076 30934846 PMC6524369

[17] Vleck VE Bürgi A Bentley DJ. The consequences of swim, cycle, and run performance on overall result in elite Olympic distance triathlon. Int J Sports Med. (2006) 27:43–8. 10.1055/s-2005-837502 16388441

[18] Vleck VE Bentley DJ Millet GP Bürgi A. Pacing during an elite Olympic distance triathlon: comparison between Male and female competitors. J Sci and Med in Sport. (2008) 11:424–32. 10.1016/j.jsams.2007.01.006 17350889

[19] Cejuela R Cala A Pérez-Turpin JA Villa JG Cortell JM Chinchilla JJ. Temporal activity in particular segments and transitions in the Olympic triathlon. J Hum Kinetics. (2013) 36:87–95. 10.2478/hukin-2013-0009 PMC3661898 23717358

[20] Millet G Millet G Candau R. Duration and seriousness of running mechanices alterations after maximal cycling in triathlets: influence of the performance level. J Sports Med and Phy Fitness. (2001) 41:147. 11447354

[21] Weich C Jensen RL Vieten M. Triathlon transition study: quantifying differences in running movement pattern and precision after bike-run transition. Sports Biomechanics. (2019) 18:215–28. 10.1080/14763141.2017.1391324 29141506

[22] Bonacci J Saunders PU Alexander M Blanch P Vicenzino B. Neuromuscular control and running economy is preserved in elite international triathletes after cycling. Sports Biomech. (2011) 10:59–71. 10.1080/14763141.2010.547593 21560752

[23] Hue O Le Gallais D Boussana A Chollet D Prefaut C. Ventilatory responses during experimental cycle-run transition in triathletes: Med & Sci in Sports & Exer. (1999) 31:1422. 10.1097/00005768-199910000-00010 10527314

[24] Millet GP Dréano P Bentley DJ. Physiological characteristics of elite short- and long-distance triathletes. Eur J Appl Physiol. (2003) 88:427–30. 10.1007/s00421-002-0731-0 12527973

[25] Bonacci J Green D Saunders PU Blanch P Franettovich M Chapman AR Vicenzino B. Change in running kinematics after cycling are related to alterations in running economy in triathletes. J Sci and Med in Sport. (2010) 13:460–4. 10.1016/j.jsams.2010.02.002 20359948

[26] Millet GP Millet GY Hofmann M Candau R. Alterations in running economy and mechanics after maximal cycling in triathletes: influence of performance level. Int J Sports Med. (2000) 21:127–32. 10.1055/s-2000-8866 10727074

[27] Chapman AR Vicenzino B Hodges PW Blanch P Hahn AG Milner TE. A protocol for measuring the direct effect of cycling on neuromuscular control of running in triathletes. J Sports Sci. (2009) 27:767–82. 10.1080/02640410902859100 19437184

[28] Karniel A Mussa-Ivaldi FA. Does the motor control system use multiple models and context switching to cope with a variable environment? Exp Brain Res. (2002) 143:520–4. 10.1007/s00221-002-1054-4 11914799

[29] Gohlitz D Große S Witt M. “Darstellungen von veränderungen der schrittlänge und schrittfrequenz beim Übergang vom radfahren zum laufen zur kennzeichnung der dauer von Übergangsphasen im duathlon (pilotuntersuchung).,” In: Engelhardt M Franz B Neumann G Pfützner A, editors. Triathlon – medizinische und methodische probleme des trainings. Triathlon und Sportwissenschaft. Hamburg: Czwalina (1994). p. 131–6.

[30] Sterzing T Brauner T Milani TL. Laufen: barfuß vs. Schuh–kinetische und kinematische adaptationen der unteren extremität. Biomechanik–Grundlagenforschung und Anwendung. (2009) 4:26–32.

[31] Connick MJ Li F-X. Prolonged cycling alters stride time variability and kinematics of a post-cycle transition run in triathletes. J Electromyogr and Kinesiol. (2015) 25:34–9. 10.1016/j.jelekin.2014.08.009 25282575

[32] Vieten MM Sehle A Jensen RL. A novel approach to quantify time series differences of gait data using attractor attributes. PLoS ONE. (2013) 8:e71824. 10.1371/journal.pone.0071824 23951252 PMC3737194

[33] Weich C. The attractor method and its application in running, bicycling and nordic skiing. [Dissertation] Konstanz: University of Konstanz. (2021). 796 p. http://nbn-resolving.de/urn:nbn:de:bsz:352-2-1op56v75zqhjm0

[34] Haworth J Walsh M Strang A Hohl J Spraets S Wilson M Brown C. Training for the bike to run transition in triathlon. In ISBS-Conference Proceedings Archive. (2010). Available from: https://ojs.ub.uni-konstanz.de/cpa/article/view/4442/4131

[35] Hue O Valluet A Blonc S Hertogh C. Effects of multicycle-run training on triathlete performance. Res Q Exerc and Sport. (2002) 73:289–95. 10.1080/02701367.2002.10609022 12230335

[36] Walsh J Peoples G Lepers R Stamenkovic A Stapley P. Activation patterns of leg muscles in trained triathletes are not variable during the early period of running after cycling. Australian Biomechanics Conference (ABC9) 2014. (2014) 10.13140/2.1.1632.4164

[37] Vleck V Alves FB. Triathlon Transition Tests: Overview and Recommendations for Future Research. *RICYDE Revista Internacional de Ciencias del Deporte doi: 105232/ricyde* (2011) 7:I–III.

[38] Vleck V Millet GP Alves FB Bentley DJ. Reliability and validity of physiological data obtained within a cycle-run transition test in age-group triathletes. (2012)9. PMC3763322 24150086

[39] Bentley D Delextrat A Vleck V Reid AK. Reliability of a sequential running-cycling-running test in trained triathletes. J Sports Sci. (2005) 23:202–10.

[40] Díaz V Peinado AB Vleck VE Alvarez-Sánchez M Benito PJ Alves FB Calderón FJ Zapico AG. Longitudinal changes in response to a cycle-run field test of young Male national “talent identification” and senior elite triathlon squads. J Strength and Cond Res. (2012) 26:2209–19. 10.1519/JSC.0b013e31823a3c6b 21997447

[41] Vleck V Santos S Bentley D Alves F. Influence of prior cycling on the OBLA measured during incremental running in triathletes. (2005). p. 93–223

[42] Evans SA James D Rowlands D Lee JB. Differences in torso kinematics between ergometer cycling and outdoor cycling in triathletes - A preliminary study. ISBS Proceedings Archive. (2021). p. 5 https://commons.nmu.edu/cgi/viewcontent.cgi?article=2273&context=isbs

[43] Alves M Vleck V Alves FB. Influence of event distance specialisation on performance within a sequential running-cycling-running test in age-group triathletes. *Proceedings of the 13th Annual Congress of the European College of Sports Science*. Estroil (Portugal) (2008)

[44] McDevitt S Hernandez H Hicks J Lowell R Bentahaikt H Burch R Ball J Chander H Freeman C Taylor C Wearables for biomechanical performance optimization and risk assessment in industrial and sports applications. Bioeng. (2022) 9:33. 10.3390/bioengineering9010033 PMC8772827 35049742

[45] Zhang X Shan G Wang Y Wan B Li H. Wearables, biomechanical feedback, and human motor-Skills’ learning & optimization. Appl Sci. (2019) 9:226. 10.3390/app9020226

[46] Sharma AP Périard JD. Physiological requirements of the different distances of triathlon. In: Migliorini S, editor. Triathlon medicine. Cham: Springer International Publishing (2020). p. 5–17 10.1007/978-3-030-22357-1_2

[47] Cuba-Dorado A Vleck V Álvarez-Yates T Garcia-Garcia O. Gender effect on the relationship between talent identification tests and later world triathlon series performance. Sports. (2021) 9:164. 10.3390/sports9120164 34941802 PMC8704964

[48] Weich C Vieten MM Jensen RL. Transient effect at the onset of human running. Biosensors. (2020) 10:117. 10.3390/bios10090117 32911677 PMC7559896

[49] Smith D Lee H Pickard R Sutton B Hunter E. Power demands of the cycle leg during elite triathlon competition. Cahiers de l’INSEP. (1999) 24:224–30.

[50] Silder A Gleason K Thelen DG. Influence of bicycle seat tube angle and hand position on lower extremity kinematics and neuromuscular control: implications for triathlon running performance. J Appl Biomech. (2011) 27:297–305. 10.1123/jab.27.4.297 21896955

[51] Munro C Miller D Fuglevand A. Ground reaction forces in running: a reexamination. J Biomech. (1987) 20:9. 10.1016/0021-9290(87)90306-X 3571295

[52] Vieten MM Weich C. The kinematics of cyclic human movement. PLoS ONE. (2020) 15:e0225157. 10.1371/journal.pone.0225157 32134925 PMC7058299

[53] Lepers R Bigard AX Diard J-P Gouteyron J-F Guezennec CY. Posture control after prolonged exercise. Euro J Appl Physiol. (1997) 76:55–61. 10.1007/s004210050212 9243170

[54] Broscheid K-C Dettmers C Vieten M. Is the limit-cycle-attractor an (almost) invariable characteristic in human walking? Gait Posture. (2018) 63:242–7. 10.1016/j.gaitpost.2018.05.015 29778064

[55] Byrnes SK Nüesch C Loske S Leuenberger A Schären S Netzer C Mündermann A. Inertial sensor-based gait and attractor analysis as clinical measurement tool: functionality and sensitivity in healthy subjects and patients with symptomatic lumbar spinal stenosis. Front Physiol. (2018) 9:1–8. 10.3389/fphys.2018.01095 30154731 PMC6102665

[56] Weich C Vieten M. The gaitprint: identifying individuals by their running style. Sensors. (2020) 20:3810. 10.3390/s20143810 32650424 PMC7412195

[57] Witt M. “Biomechanische untersuchungen zum belastungswechsel im triathlon.,” In: Engelhardt M. Franz B. Neumann G. Pfützner A., editors. Triathlon – medizinische und methodische probleme des trainings. Triathlon und Sportwissenschaft. Hamburg: Czwalina (1994).

[58] Hausswirth C Le Meur Y Bieuzen F Brisswalter J Bernard T. Pacing strategy during the initial phase of the run in triathlon: influence on overall performance. Eur J Appl Physiol. (2010) 108:1115–23. 10.1007/s00421-009-1322-0 20024576

[59] Skroce K Tarperi C Brasi I Bertinato L Schena F. Fast or slow start? The role of running strategies in triathlon. J Sci and Med in Sport. (2022) 25:70–4. 10.1016/j.jsams.2021.07.013 34446367

[60] Meur YL Bernard T Dorel S Abbiss CR Honnorat G Brisswalter J Hausswirth C. Relationships between triathlon performance and pacing strategy during the run in an international competition. Int J Sports Physiol and Perf. (2011) 6:183–94. 10.1123/ijspp.6.2.183 21725104

