# Supplementary material for: Discovering the sluggishness of triathlon running - using the attractor method to quantify the impact of the bike-run transition
Source: Front Sports Act Living. 2022 Dec 16;4:1065741. doi: 10.3389/fspor.2022.1065741 (PMC9802668; doi:10.3389/fspor.2022.1065741)
Supplement: Supplementary file 1 [file Datasheet1.pdf]

Subject number	Gender	Age (years)	Height (cm)	Weight (kg)	Body fat (%)	Triathlon experience (years)	Threshold pace (km/h)	Vo2max run (ml/min/kg)	Threshold power (watt)	Vo2max bike (ml/min/kg)	Transition time (Min)
1	m	31	178	70	10	10	15.1	59.3	266	63.9	1.7
2	f	25	157	51	18	4	12.7	47.2	189	55.8	5.6
3	m	27	178	70.7	10	5	15.5	62.9	269	64.4	2.1
4	m	22	170	70	16	4	11.7	55.9	210	52.6	1.5
5	m	25	180	68.9	8	4	15.3	66.4	241	58.2	0.9
6	m	24	175	69	12	3	15.3	63.2	290	65.5	0.9
7	m	26	178	72	18	2	15.8	64.8	245	51	1.3
8	m	32	190	88	18	3	13.6	59	273	50	2.8
9	m	28	176	73.5	12.8	7	14.6	58.22	272	54.51	1.8
10	m	45	176	72	8	20	12.4	52.3	259	56.2	1.9
11	m	23	186	70.4	8	3	14.9	66.4	253	59.7	2.5
12	m	28	183	73	10	9	14.8	60.4	299	68	4
13	f	54	170	58	23	3	9.5	39.7	209	52.1	1.9
14	f	30	169	57	18	4	12	51.6	160	49	4.8
15	f	31	166	60	19	2	12.3	52.8	204	46	2.7
16	f	31	179	75	25	2	11.9	47.3	233	47.6	2.2
17	f	39	165	58.8	21	4	13.1	52.6	200	51.9	4.3
18	f	32	173	69	15	14	13.5	53	242	52.7	4.3
19	m	23	179	70	9	12	16.3	63.9	331	72.2	2.8
20	f	25	160	59.3	22	13	12	56	190	55.4	2.5
21	f	23	165	55	20	1	13.4	51.4	165	47.1	2.7
22	f	21	174	59.7	18.4	4	12.3	48.4	155	44.1	2.1
